# Should I stay or should I go? Conceptual underpinnings of goal-directed actions

**DOI:** 10.3389/fnsys.2014.00206

**Published:** 2014-11-03

**Authors:** Giovanni Mirabella

**Affiliations:** ^1^Istituto Neurologico Mediterraneo, IRCCS Neuromed, PozzilliItaly; ^2^Department of Physiology and Pharmacology ‘V. Erspamer,’ La Sapienza University, RomeItaly

**Keywords:** decision-making, reward, voluntary motor control, behavioral flexibility, countermanding task, reaching arm movements

## Abstract

All actions, even the simplest like moving an arm to grasp a pen, are associated with energy costs. Thus all mobile organisms possess the ability to evaluate resources and select those behaviors that are most likely to lead to the greatest accrual of valuable items (reward) in the near or, especially in the case of humans, distant future. The evaluation process is performed at all possible stages of the series of decisions that lead to the building of a goal-directed action or to its suppression. This is because all animals have a limited amount of energy and resources; to survive and be able to reproduce they have to minimize the costs and maximize the outcomes of their actions. These computations are at the root of behavioral flexibility. Two executive functions play a major role in generating flexible behaviors: (i) the ability to predict future outcomes of goal-directed actions; and (ii) the ability to cancel them when they are unlikely to accomplish valuable results. These two processes operate continuously during the entire course of a movement: during its genesis, its planning and even its execution, so that the motor output can be modulated or suppressed at any time before its execution. In this review, functional interactions of the extended neural network subserving generation and inhibition of goal-directed movements will be outlined, leading to the intriguing hypothesis that the performance of actions and their suppression are not specified by independent sets of brain regions. Rather, it will be proposed that acting and stopping are functions emerging from specific interactions between largely overlapping brain regions, whose activity is intimately linked (directly or indirectly) to the evaluations of pros and cons of an action. Such mechanism would allow the brain to perform as a highly efficient and flexible system, as different functions could be computed exploiting the same components operating in different configurations.

## INTRODUCTION

Our survival depends on the ability to gather, parse and evaluate the stream of constantly changing environmental stimuli and to flexibly adapt our behavioral responses according to the context in which we are embedded. This is because all animals operate with limited resources, and thus the way they value their internal states, sensory experience, and behavioral output influences directly how they will invest their time and energy (Rangel et al., 2008). The bottom line is that the opportunity of executing any action needs to be continuously evaluated in order to minimize its costs and to maximize its payoffs. In fact, the value associated with a certain stimulus is not an intrinsic property of the stimulus, but can change as a function of the internal states of the agent at the time the stimulus is encountered and as a function of agent’s previous experience with that stimulus. For instance, for a thirsty gazelle the water of a pond might represent a highly valuable stimulus, unless it perceives the presence of hungry lions.

Central to this process are two executive functions: (i) the ability to predict the future outcomes of a given action; and (ii) the ability to suppress inappropriate, i.e., not sufficiently valuable, actions. Importantly, these two executive functions operate not only during the genesis of an action, but also during the planning of an already selected action. In fact, during the temporal gap between the time when an action has been chosen and the moment when the motor output is going to be generated, the context might have changed, altering the computed value of the action and thus requiring a radical change of the planned motor strategy. For instance, the sight of a tasty cake is likely to drive a child to plunge his fingers into the cream, but if, when he is about to act, he suddenly feels he is observed by his parents, he will refrain from executing the planned movement. In this instance, the fear of being punished has overcome the potential reward of a sweet food, causing the suppression of the pending action.

**FIGURE 1:**
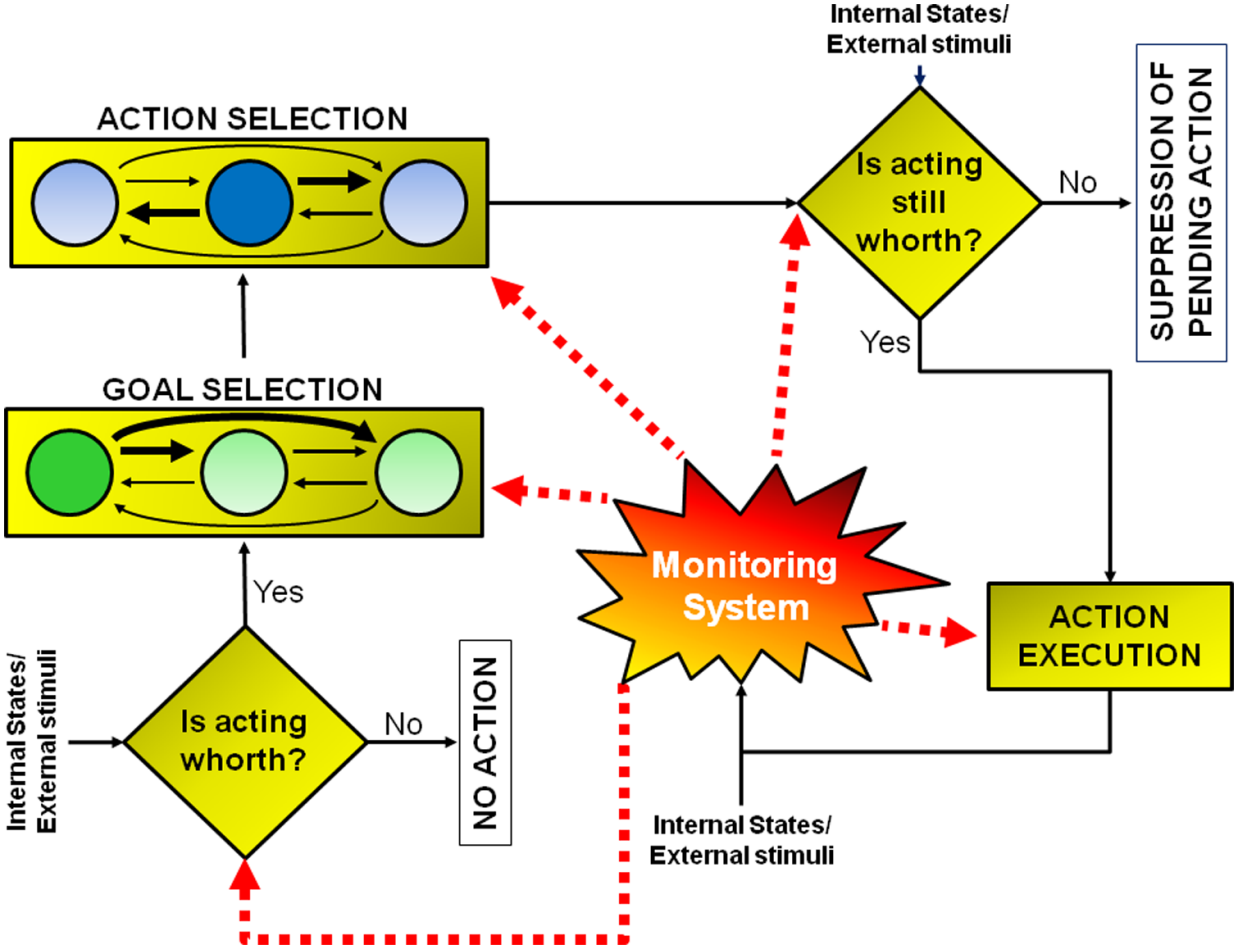
**Model of goal-directed actions.** The model consists of a set of a multi-step decision processes leading either to the execution or to the inhibition of an action, according to the evaluation of its pros and cons. This model does not have a strictly serial or parallel architecture. Some processes must occur before others (e.g., the early “should-I-stay-or-should-I-go” decision aimed at evaluating whether acting is worthwhile must occur before goal or action selection), but other processes occur in parallel (e.g., the monitoring system, whose role is to compute predictions about future reward and to measure discrepancies between expected and actual outcomes, is active during all the steps). See text for further details.

Conceptually, a goal-directed action can be modeled as multi-step decision process to which several brain regions contribute (see **Figure 1**; **Table 1**). The different stages leading up to the execution or the inhibition of an action are described in the next sections. The model I propose has been inspired by that suggested by Haggard (2008). However, there are two key differences between the former and the latter. First of all, the new model does not necessarily subserve human volition (see paragraph ‘Concluding Thoughts’). Secondly, in this new model, each stage of the process can be influenced by a component named monitoring system, according to the results of the outcomes of previous decisions (see paragraph ‘The Monitoring System’). As a consequence the model I propose does not work strictly in a serial fashion, but both serially and in parallel.

**Table 1:** Summary of the main brain regions involved in each stage of the model describing the genesis of an arm goal oriented action, together with the most relevant references.

**Early “should-I-stay-or-should-I-go” decision**	SMA/pre-SMA	Boccardi et al. (2002), Grezes and Decety (2002), Biran and Chatterjee (2004), Sumner et al. (2007), Forstmann et al. (2008), Ridderinkhof et al. (2011), van Gaal et al. (2011)
	Sector F5 (subregion of PMv)/PPC	Murata et al. (1997), Rizzolatti and Luppino (2001)
	Basal ganglia (dopaminergic neurons; putamen and pallidum)	Mazzoni et al. (2007), Schmidt et al. (2008), Bonanni et al. (2010), for a review see Turner and Desmurget (2010)
**Goal selection**	OFC	Damasio (1994), Padoa-Schioppa and Assad (2006), Fellows and Farah (2007), Padoa-Schioppa and Assad (2008), for a review see Wallis (2007)
	LPFC	Hoshi et al. (2000), Barraclough et al. (2004), Genovesio et al. (2005, 2012), for a review see Wise (2008)
**Action selection**	PMd	Cisek and Kalaska (2005), Klaes et al. (2011), for a review see Cisek and Kalaska (2010)
	PRR	Calton et al. (2002), Cui and Andersen (2007), Scherberger and Andersen (2007)
**Late “should-I-stay-or-should-I-go” decision**	IFG/DLPFC (subregions of LPFC)	Aron et al. (2003), Aron and Poldrack (2006), Chambers et al. (2006), Aron et al. (2007), Zheng et al. (2008), Zandbelt and Vink (2010), Jahfari et al. (2012), Zandbelt et al. (2013), for a review see Aron et al. (2014)
	Pre-SMA	Aron et al. (2007), Li et al. (2008), Chen et al. (2010), Zandbelt and Vink (2010), Jahfari et al. (2012), Zandbelt et al. (2013)
	Basal ganglia (striatum, STN)	Aron and Poldrack (2006), Van den Wildenberg et al. (2006), Aron et al. (2007), Li et al. (2008), Zandbelt and Vink (2010), Swann et al. (2011), Mirabella et al. (2012)
	PMd and M1	Coxon et al. (2006), Swann et al. (2009), Mirabella et al. (2011), Mattia et al. (2012), Mattia et al. (2013)
	PPC	Chikazoe et al. (2009), Jahfari et al. (2010), Zandbelt et al. (2013)
**Action execution**	PMd and M1	Tanji and Evarts (1976), Georgopoulos et al. (1982), Weinrich et al. (1984), Riehle and Requin (1989), Hoshi and Tanji (2000), Hoshi and Tanji (2002), Churchland et al. (2006), for a review see Shenoy et al. (2013)
	Spinal cord	Prut and Fetz (1999)
	Basal ganglia (STN)	Paradiso et al. (2003), Loukas and Brown (2004), Mirabella et al. (2013)
**Monitoring system**	ACC	Bernstein et al. (1995), Brown and Braver (2005), Kennerley et al. (2006), Sheth et al. (2012)
	Basal ganglia (dopaminergic neurons; ventral striatum)	Schultz et al. (1997), O’Doherty et al. (2003), Montague et al. (2004), Bayer and Glimcher (2005), Chikazoe et al. (2009), Zandbelt and Vink (2010), Jahfari et al. (2012), Zandbelt et al. (2013)
	Frontal pole cortex (subregion of PFC)	Tsujimoto et al. (2010), for a review see Tsujimoto et al. (2011)
	SMA/pre-SMA	Chikazoe et al. (2009), Chen et al. (2010), Scangos and Stuphorn (2010), Zandbelt and Vink (2010), Jahfari et al. (2012), Zandbelt et al. (2013), Bonini et al. (2014)

*SMA, supplementary motor area; pre-SMA, pre-supplementary motor area; PMv, ventral premotor cortex; PPC, posterior parietal cortex; LPFC, lateral prefrontal cortex; DLPFC, dorsolateral prefrontal cortex; PMd, dorsal premotor cortex; PRR, parietal reach region; IFG, inferior frontal gyrus; STN, subthalamic nucleus; M1, primary motor cortex; ACC, anterior cingulate cortex; FPC, frontal pole cortex.*

Before proceeding, I want to remark that I will mostly deal with upper limb action control, leaving aside saccadic eye movement control. This is because the saccadic system has a different functional organization from that controlling arm and hand movements (e.g., see Churchland and Shenoy, 2007; Schall and Godlove, 2012). For instance, a number of studies have provided evidence that the inhibitory controls of eye and hand movements are independent (e.g., Logan and Irwin, 2000; Boucher et al., 2007; Sumner et al., 2007; Mirabella et al., 2011). Interestingly, Mirabella et al. (2009) and Stevenson et al. (2009) studied the effect of introducing a temporal gap between the end of the fixation and target presentation on the reaction times (RTs) and on the speed of inhibition of reaching movements and saccades, respectively. They found contrasting results: in the former study the gap produced a decrease in the inhibitory speed and in the latter an increase. This is not odd, as saccades have a different ecological relevance from hand and arm movements in primates. In fact, outside neurophysiology laboratories, they allow physical interactions with the environment, thus leading to material outcomes such as acquisition of food or tools. Nevertheless, it is likely that the very general principles, not the fine details, of the genesis of eye and limb movements are rather similar.

### THE MOTIVATION TO ACT (EARLY “SHOULD-I-STAY-OR-SHOULD-I-GO” DECISION)

The first step is represented by the motivation to act (early “should-I-stay-or-should-I-go” decision). This is determined by an evaluation process which is aimed at determining whether or not the individual’s current needs are satisfied. The evaluative process can be primed because of a change in either the external environment (e.g., the sight of a cake) or the internal states (e.g., a sudden hungry feeling), or both (e.g., the sight of a cake prompts a hungry feeling). Thus, these first computations would evaluate whether or not the current state has to be changed to pursue a desire (e.g., eating) against several possible constraints (e.g., eating too much might cause weight gain). If the motivation is considered worthwhile then movement preparation will jump to the next stage, otherwise it will be canceled.

**FIGURE 2:**
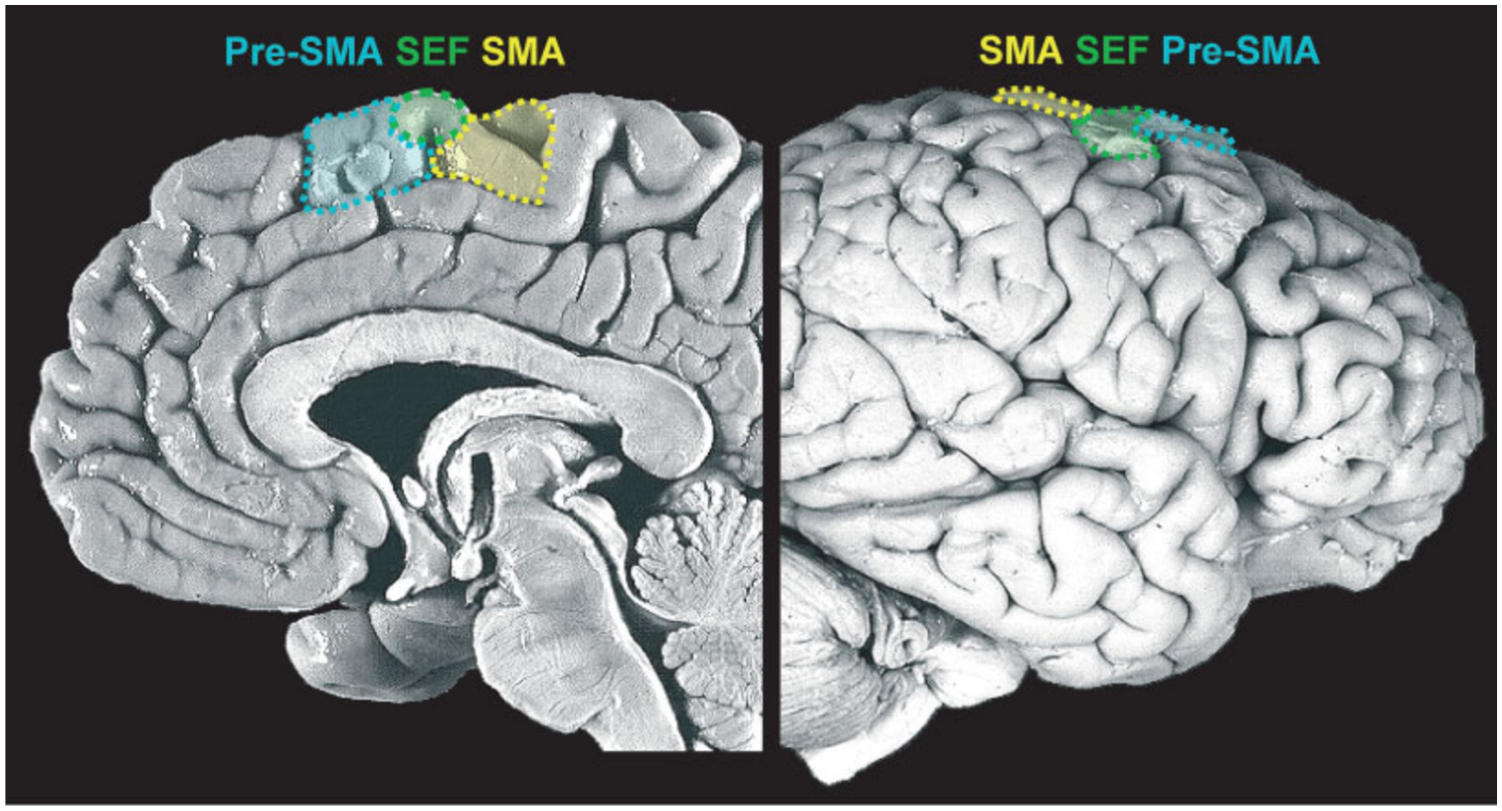
**Medial frontal cortex (details of the medial portion of Brodmann areas 6 and 8).** Midsagittal view of the medial wall **(left)** and lateral prefrontal cortex (LPFC) surface **(right)**, delineating the supplementary motor area (SMA), supplementary eye field (SEF) and pre-supplementary motor area (pre-SMA). Reproduced with permission from Ridderinkhof et al. (2011).

This is an essential process and one which is continuously performed by our motor system. In fact, in most places where we live, if not all, we are surrounded by tools whose sight automatically activates motor schemas that would normally be employed to interact with those objects. These actions are prompted by the features of the objects, the so-called affordances (Gibson, 1979). It has been shown that even the simple observation of pictures depicting affordable objects (such as graspable objects) activates a sub-region of the medial frontal cortex, the supplementary motor area (SMA; see **Figure 2**), even when there is no requirement to actually act on those stimuli (Grezes and Decety, 2002). These stimulus-driven activations are rapid, involuntary, and unconscious.

Sumner et al. (2007), using subliminal stimuli to prime movements in a direction opposite to the requested one, showed that while control subjects were able to withhold unwanted actions, patients with microlesions of the SMA or the supplementary eye fields (SEFs) were impaired during the execution of hand or eye movements, respectively. They concluded that the SMA and SEF mediate automatic effector-specific suppression of motor plans. However, there is another way to look at those automatic activations: they might represent the activity of the network subserving the evaluative process which is at the base of the early “should-I-stay-or-should-I-go” decision. In other words, affordances might increase the motivation to act, but to execute an action they have to be coupled with an internal state congruent with the primed action (e.g., the sight of a glass of water will prime the action if and only if an individual is thirsty; see **Figure 1**).

From this perspective the suppression of a triggered action might be seen not as an active process, but rather as an automatic consequence of the evaluative procedure. Using this framework, is possible to put forward a functional hypothesis underling two rare neuropsychological disorders, the alien limb syndrome and the utilization behavior. Both diseases are characterized by the fact that patients cannot resist objects’ affordance, and they are automatically forced to perform stimulus-driven motor responses even when they do not need those objects (Humphreys and Riddoch, 2000). Patients with the alien hand syndrome perform involuntary actions with the limb contralateral to a focal brain lesion most frequently located in the medial frontal cortex, usually involving the SMA (Della Sala et al., 1991; Biran and Chatterjee, 2004). Patients suffering from utilization behavior compulsively grasp and use objects placed within their reach. This syndrome has been linked to bilateral damage to the medial frontal region involving the SMA, pre-SMA, and cingulate motor areas (Boccardi et al., 2002). Therefore, at least to some extent, the sites of the lesions causing those syndromes are largely overlapping, with the difference that in the former case it is located just in one hemisphere whereas in the latter case it affects both hemispheres. Possibly what is affected in both syndromes is the circuitry underlying the early “should-I-stay-or-should-I-go” evaluation, so that most stimulus-driven activations are no longer matched with our internal needs and thus they cannot be filtered out.

The discovery of the so-called canonical neurons in monkeys’ lateral premotor area F5 may represent the neural mechanisms underlying responsiveness to object affordances (Murata et al., 1997). These neurons become active both when grasping an object and when seeing the same object without moving (Murata et al., 1997), and they are likely to feed the neural network of the medial frontal region which presides over the evaluation of whether to act. In fact, the lateral premotor area F5 receives projections from several regions of the posterior parietal cortex (PPC; Rizzolatti and Luppino, 2001), which is the end point of a crucial pathway for the visual guidance of actions toward objects, named the dorsal stream (Milner and Goodale, 1995). As such, PPC and F5 are likely to play a key role in visuomotor transformations, providing a neurophysiological correlate of stimulus-driven action affordance.

Several lines of evidence indicate that the fate of these activations might be decided in pre-SMA, which would act as a gate through which the available action affordances might be translated into actual actions (Ridderinkhof et al., 2011). First of all, a functional magnetic imaging (fMRI) study has shown that the strength of activation in pre-SMA covaries with the extent of inappropriate responses driven by stimulus-action association, i.e., the selection of appropriate action engages stronger activation of the pre-SMA in the face of many competing alternatives (Forstmann et al., 2008). Second, a strong negative correlation has been demonstrated between pre-SMA gray-matter volume and the inability to efficiently deal with competing response tendencies (van Gaal et al., 2011).

It would be very reductive to limit this first stage to the filtering of stimulus-driven action affordances and to the medial frontal regions. In fact, a role is surely also played by those brain regions that underlie arousal regulation (in particular the dopaminergic system), increasing or decreasing the readiness of animals to react. There are a wide variety of stimuli that can trigger arousal, from relatively simple sensory stimuli (e.g., the smell of blood or the sound of a mating call) to much more complex situations (e.g., the feeling of social exclusion or the feeling of being idolized). These signals prompt some basic instincts or learned memories which, according to the current contextual situation, might change the internal state of the animals, triggering an alert state. In these instances, animals do not yet have a goal, but they are more ready to act. The dopaminergic system is likely to play an important role in this stage of action genesis as witnessed by one of the symptoms of the most severe form Parkinson’s disease (PD), the akinesia, i.e., the inability to initiate any goal-directed movement. Akinesia is not a pure motor disturbance, because it has been shown that in situations of great emergency (e.g., a missile attack, an earthquake) otherwise akinetic PD patients can show a sudden transient ability to move (paradoxical kinesia; Glickstein and Stein, 1991; Schlesinger et al., 2007; Bonanni et al., 2010). Evidently, when dopamine neurons are remarkably reduced in number, all behavioral options or internal mental states would appear to have the same value as the current state. In the face of this flat value function, the best choice for PD patients is to freeze. In extreme situations, salient stimuli may elicit stronger discharge of dopaminergic cells than normal, driving the patient to act. Recently, Mazzoni et al. (2007) suggested that even in less dramatic situations the slowness of movements (bradikinesia) in PD patients may be attributable to an improper evaluation of movement energy costs. In these experiments, PD patients and healthy controls were asked to move their arm to a previously specified target at different speeds a given number of times. Both were able to make required movements with the same accuracy, but PD patients needed significantly more trials before reaching the required number of repetitions. As the accuracy of patients was the same as that of controls, authors concluded that the loss of dopamine did not cause bradykinesia through a speed-accuracy trade-off. Rather, it affected decision-making through a faulty evaluation of the costs of movements, i.e., allocation of the correct amount of energy to meet the demands of the task. In other words, PD weakened the key link between motivation and movement gain. In fact, the dopaminergic system has been shown to play a major role in reward-dependent learning (e.g., Schultz et al., 1997; Montague et al., 2004).

Other evidence indicates that the regulation of action motivation based on previous experiences is one of the main functions of the basal ganglia, not just of the dopaminergic system (Turner and Desmurget, 2010). This hypothesis is supported by one symptom which often accompanies focal damage of the basal ganglia, the so-called auto-activation deficit or abulia (Habib, 2004), in which patients suffer from a marked deficit in motivation to perform spontaneous acts despite an absence of overt motor impairment. In particular, Schmidt et al. (2008) asked patients suffering from auto-activation deficit, due to bilateral lesions of the putamen or pallidum, to control grip forces in response either to explicit sensory instructions or to monetary incentives. Although they fully understood the instructions, patients were capable of modulating their movement only in the former, not in the latter, condition.

All in all, it is clear that there are several brain regions that regulate the willingness to act. This early “should-I-stay-or-should-I-go” decision should play a key role because it would activate (or stop) the chain of other decisions that will potentially lead to action execution. While many other subsequent stages might be performed in parallel, this first one is likely to be a stand-alone process during which no actions are planned and thus there is no need for any neural signal to inhibit them.

### GOAL SELECTION

**FIGURE 3:**
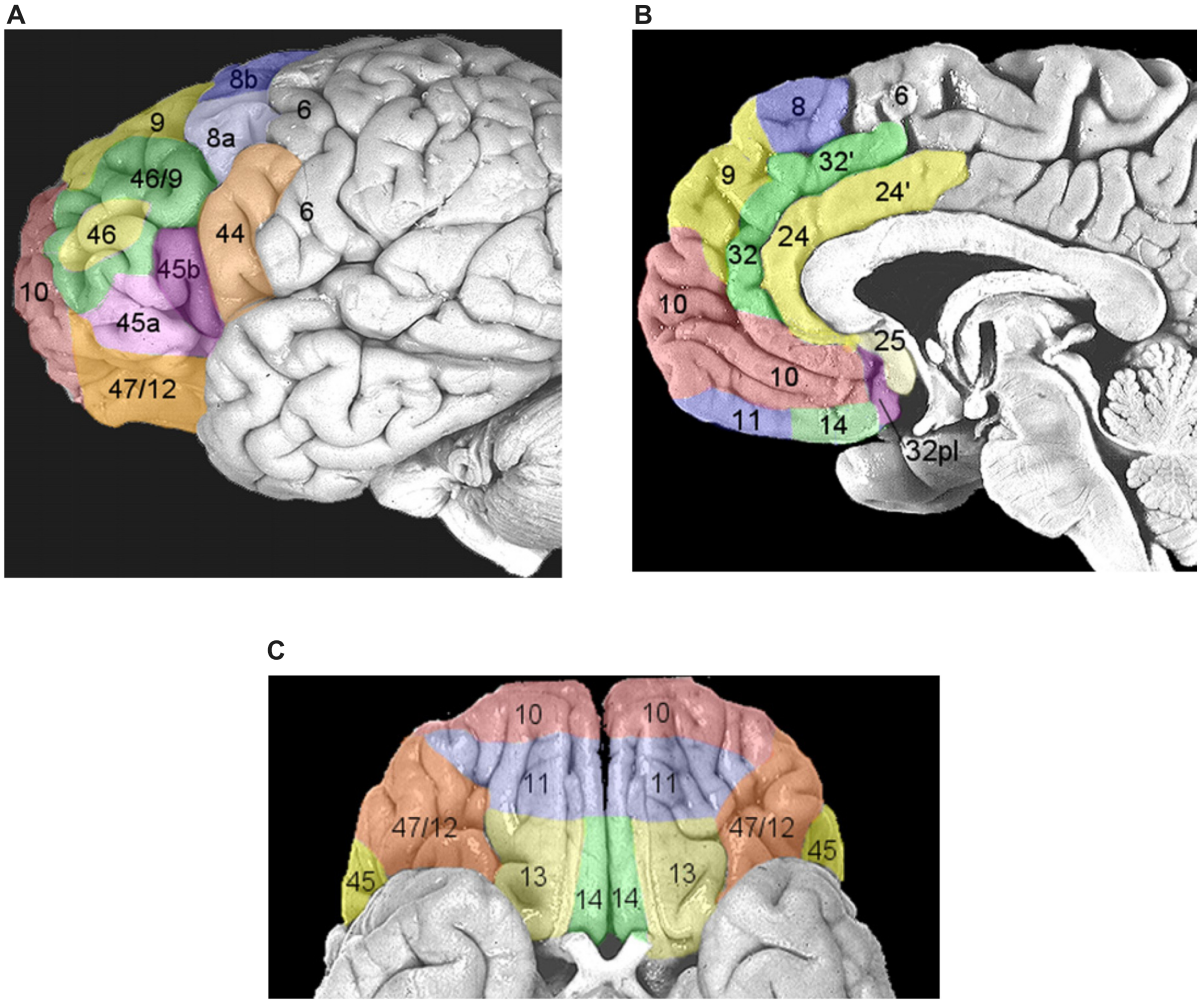
**Areas composing the prefrontal cortex (PFC) according to the parcellation of Petrides and Pandya (1999, 2002). (A)** Areas of the lateral PFC. The inferior frontal gyrus (IFG) corresponds to Brodmann area (BA) 44, or pars opercolaris, to BA45, or pars triangularis, and to BA47/12, or pars orbitalis. The dorsolateral prefrontal cortex (DLPFC) corresponds to BA9/46 and BA46. **(B)** Areas of the medial wall of the PFC. The anterior cingulate cortex (ACC) corresponds to BA32, BA24, and BA25 (dorsocaudal portions are indicated with a hyphen). BA10 corresponds to the frontopolar cortex. The orbitofrontal cortex (OFC) corresponds to BA11 and BA14. **(C)** Areas of ventral orbital surface of the PFC. BA10 corresponds to the frontopolar cortex. Area BA47/12 corresponds to the pars orbitalis of the IFG. The OFC corresponds to BA11, BA13, and BA14. Reproduced with permission from Ridderinkhof et al. (2004).

Once performance of an action is considered worthwhile, because a need has to be satisfied, the next set of decisions will be devoted to the selection of the most opportune goal among the several different alternatives usually available (e.g., to satisfy the feeling of hunger we might decide to eat the cake in front of us or to leave the room and go to a restaurant). This process possibly entails two stages: first, values are assigned to each available option and subsequently a decision is taken weighting these values according to the behavioral context (e.g., Glimcher et al., 2005). Even though this schema is conceptually logical, it is very unlikely that neural processing would be strictly organized in distinct serial stages. As depicted in **Figure 1**, it is very likely that multiple potential goals are simultaneously represented. To each goal a given value would be assigned and weighted according to the situation. As multiple goals cannot be pursued at the same time, these representations probably start to compete with each other, perhaps through mutual inhibition (**Figure 1**). This competition can be biased by several factors which ultimately influence the expected outcome of the action^1^. In this review I will argue that these biasing signals might come from the brain regions of the “monitoring system,” i.e., the system that evaluates and stores the outcome of past actions (see **Figure 1** and see The Monitoring System). Value assignment and the competition might occur at the same time, probably in different sectors of the prefrontal cortex (PFC). PFC is not a homogeneous structure but is composed of multiple areas that differ in terms of cytoarchitecture and anatomical connections with other areas (Petrides and Pandya, 1999, 2002; **Figure 3**). Thus, it is likely that each area might perform its own function; however, their exact roles are not yet clear. Part of the reason is that the PFC contributes to a bewildering array of functions (Wise, 2008).

Nevertheless, converging evidence argues that orbitofrontal cortex (OFC) plays a key role in linking stimuli to their values (for a review see Wallis, 2007). Indeed, lesions to the OFC impair choice behavior, leading to unreliable choices (e.g., Damasio, 1994; Fellows and Farah, 2007) or abnormal gambling (e.g., Camille et al., 2004; Koenigs and Tranel, 2007). Padoa-Schioppa and Assad (2006), recording single-units in monkeys, demonstrated that a particular class of neurons of the OFC (“chosen neurons”) encode the subjective value of two different drinks (juice and water) irrespective of their taste, volume or the action that needs to be taken to obtain them. Thus these cells encoded value *per se*, allowing a comparison for qualitatively different goods. Later on, Padoa-Schioppa and Assad (2008) also showed that the discharge of these neurons was independent of the presence of other goods. These findings indicate that the OFC produces stable value representations, i.e., a key trait of choices, because it allows abstract comparisons such as transitivity between different goods which are not available at the same time.

**FIGURE 4:**
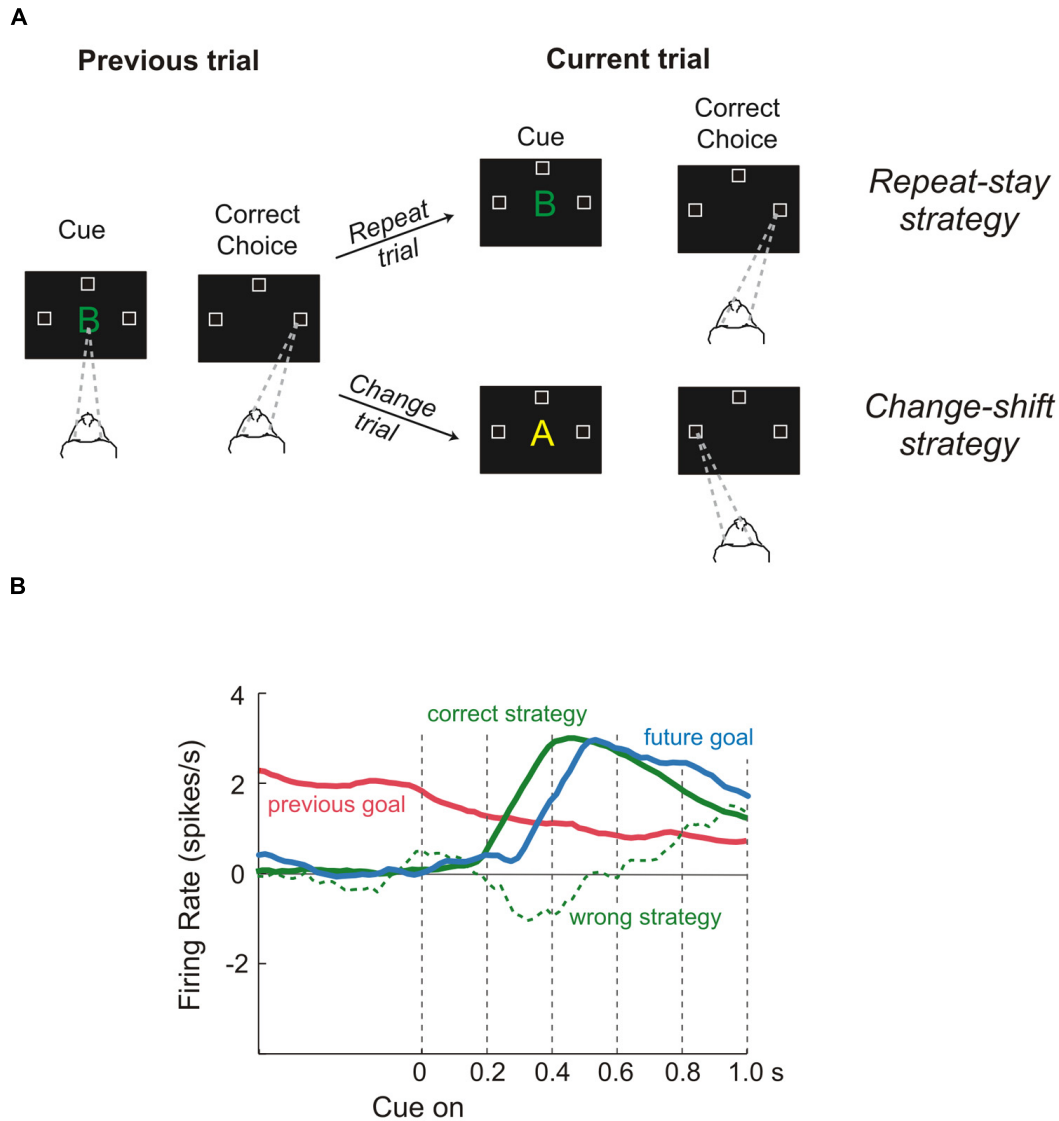
**Role of dorsolateral prefrontal cortex during a “strategy task.” (A)** Temporal sequence of the visual displays during the task and behavioral responses required of the monkeys. Each trial began when monkeys fixated on a spot at the center of the display (gaze direction is indicated by the dashed lines). After a delay, a cue appeared. When it disappeared, the monkeys had to make a saccadic eye movement to one of three positions (unfilled squares). Monkeys were required to remember both the cue and the goal of the trial just performed because the response on the next trial (current trial) depended on the previous choice, i.e., if the cue was the same as in the previous successful trial, the monkeys repeated the response (repeat trial), while if the cue was different they had to change their response (change trial). Thus, monkeys were forced to change strategy according to the past trial history, adopting either a repeat-stay strategy or a change-shift strategy **(B)** Neural activity reflecting the previous goal (red), the future goal (blue), the correct strategy (solid green line), and the wrong strategy (dashed green line). Previous-goal signal decreased after cue onset as the signals for the correct strategy and future goal increased. In contrast, when monkeys chose the wrong strategy, a weak or absent strategy signal occurred during the time of goal selection. Reproduced with permission from Wise (2008).

On the other hand, to select properly the more appropriate goal, the values of the options must be evaluated in light of the situation which an animal has to face (e.g., if an animal is starving even a non-preferred food represents a good choice). Therefore, to represent value efficiently in different situations, a neuronal representation should flexibly adapt to the current context. These computations are probably carried on in lateral prefrontal cortex (LPFC). Several studies have shown that neural activities of those areas specify the so-called task sets. A task set is a configuration of perceptual, attentional, mnemonic, and motor processes that is actively maintained to perform a given task. It specifies the rules needed to solve the specific task, but it is independent of the stimuli as long as they have to be processed in the same way. Therefore it is not surprising that the PFC contributes to an enormous array of functions ranging from selective attention (Lebedev et al., 2004) to working memory (Funahashi et al., 1989), problem-solving strategies (Genovesio et al., 2005) and categorization of sensory stimuli (Freedman et al., 2002). This list is by no means exhaustive, but it is intended to give an idea of the several kinds of knowledge that are processed in the LPFC. Recently, a series of studies has provided evidence about the way in which this array of cognitive processes can be combined to produce sophisticated behavior (Genovesio et al., 2005, 2006, 2012). The logic underlying all these experiments was that of setting tasks which require several kinds of long-term and short-term knowledge, while recording single-unit activity in the LPFC. For instance, Genovesio et al. (2005) trained monkeys to make a saccade to the left, right, or upward direction in response to a visual object, depending on the cue and on the goal that had occurred on the previous trial (**Figure 4**). This way the monkeys could not learn a fixed stimulus-response association. Instead, they had to adopt repeat-stay and change-shift strategies, i.e., if the cue was the same as in the previous successful trial the monkeys repeated the response, while if the cue was different they had to change their response. Genovesio et al. (2005) found that the activity of some single-neurons in the dorsolateral prefrontal cortex (DLPFC) represent different strategies, that is, special kinds of abstract rules acquired on the basis of task performance history. Similarly, Barraclough et al. (2004) found that DLPFC neurons encoded monkeys’ past decisions and payoffs, providing crucial signals to update estimates of expected reward. Thus, according to the authors the activity of these cells subserves the optimization of decision-making strategies. Other neurons in the same area represented fixed stimulus–response mappings learned previously (Hoshi et al., 2000).

All in all it seems that the different subregions of the LPFC process several types of knowledge and, taking into account the context in which the animals operate and the outcome of the action performed, allow the performance of non-routine, i.e., flexible, behaviors (Wise, 2008). Clearly such complex elaborations cannot be done in isolation. In fact, it has been recently proposed that information about several different metrics of available resources in the surrounding environment (e.g., numerosity, duration, distance) are provided to the LPFC by the PPC (Genovesio et al., 2014). Certainly, along the fronto-parietal network attentive signals flow bidirectionally, allowing the selection of salient stimuli during visually guided movements (e.g., Lebedev and Wise, 2001; for reviews see Corbetta and Shulman, 2002; Reynolds and Chelazzi, 2004). Two other relevant sources of information are the OFC, which, as described at the beginning of this paragraph, delivers knowledge about the value of the stimuli, and the anterior cingulate cortex (ACC) and/or the pre-SMA, which deliver signals dealing with prediction of expected outcome (see The Monitoring System). In addition, sensory areas could also contribute to this process. For instance, it has been found that activity of neurons in macaque area V4 can underlie the selection of elemental object features and their translation into a response-related format that can directly contribute to the control of the animal’s actions (Mirabella et al., 2007).

These are just a few examples; many cortical and subcortical structures are connected with the PFC, which is optimally situated to gather and synthesize information to select the more appropriate goal, and the best task set to achieve it in a given context.

### ACTION SELECTION

Even though goal and strategies are selected in the PFC, there are several lines of evidence indicating that choosing between alternative actions to achieve an identified goal and the generation of specific motor commands (i.e., motor plans) are accomplished by neural populations in the dorsal premotor cortex (PMd; Cisek and Kalaska, 2005; Klaes et al., 2011), in the primary motor cortex (Tanji and Evarts, 1976) and in the so-called parietal reach region (PRR), a subregion of the PPC (Cui and Andersen, 2007; Scherberger and Andersen, 2007). Therefore goal selection and the selection of movements to reach them are two separate processes that, to some extent, are likely to occur one after the other.

**FIGURE 5:**
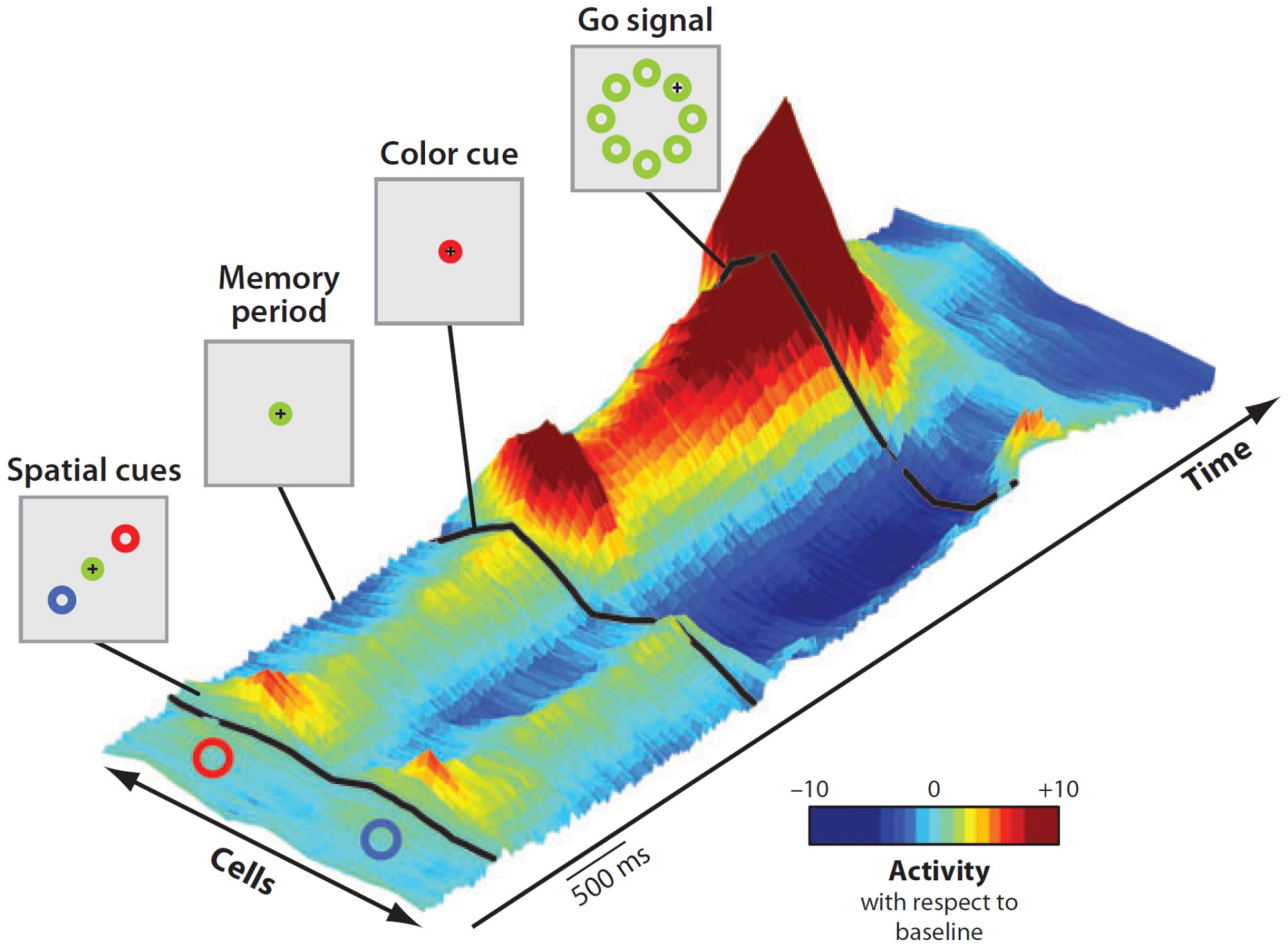
**Time course of population activity in the dorsal premotor cortex during a reach-selection task.** The diagrams on the left replicate the temporal sequence of the visual displays during the task. Each trial began with the monkeys moving the cursor (+) into a central green circle. Next, a red and a blue cue circle appeared at two of eight possible target positions, in opposite directions from the center for about a second (first display, “Spatial cues”). Then the cues disappeared (second display, “Memory period”) and after a variable period the central circle changed color to red or blue (third display, “Color cue”). Finally, the go signal was delivered (fourth display). The central circle disappeared and green circles appeared at all eight positions. To perform correctly the monkeys had to move the cursor from the central circle to the target indicated by the color cue. The 3-D colored surface on the right depicts changes of neural activity along time with respect to baseline, with cells sorted by their preferred direction along the bottom edge. Note that during the entire period of ambiguity until the presentation of the color cue, the population encoded both potential directions. When the color cue provided the information for selecting the correct action, its neural representation was strengthened while the other was suppressed. Reproduced with permission from Cisek and Kalaska (2010).

As most goals can be achieved in any of several ways, multiple potential actions are possibly represented at the same time and start to compete for implementation^2^. That this was the case was demonstrated by Cisek and Kalaska (2005) in a seminal experiment. While recording from PMd, they set a task in which two spatial cues indicated two opposite potential reaching actions. After a delay, a non-spatial cue specified the correct choice (**Figure 5**). Soon after the presentation of the two cues, the neural activity of PMd specified both directions of potential reach targets simultaneously. When information for selecting the correct action became available, its neural representation was strengthened while the other was suppressed.

On these grounds, the authors hypothesized that PMd prepares multiple actions in parallel and selects between them through a process of biased competition taking place within the same neural substrate that guides the execution of those actions (Cisek, 2006; Cisek and Kalaska, 2010). Thus, the mechanism of action selection is likely to occur in a similar way to that underlying goal selection (**Figure 1**).

Dorsal premotor cortex is not the only site where multiple potential actions can be represented. In fact, it has been shown that if a monkey is presented with a spatial target, but not instructed about whether an arm or a saccadic eye movement is required, neurons begin to discharge simultaneously in different regions of the PPC, the lateral and the medial intraparietal sulcus (LIP and MIP, respectively; Calton et al., 2002; Cui and Andersen, 2007). These discharges represent the simultaneous coding of saccade and reach plans. Later, if an arm movement is cued (Calton et al., 2002) or freely chosen (Cui and Andersen, 2007), the activity of MIP becomes stronger than that of LIP, and vice versa if a saccade is instructed or chosen.

Recently, Klaes et al. (2011) confirm and extended these findings by demonstrating that in situations of uncertain choice, the frontoparietal reach areas (PRR and PMd) construct all potential motor alternatives. However, Klaes et al. (2011) made a further step showing that that potential actions were also biased by the monkeys’ subjective desirability, confirming the model proposed by Cisek (2006).

All in all, these findings seem to indicate that in the frontoparietal reach areas there is a continuous and simultaneous processing of multiple movement options. Possibly, as some computational models predict (e.g., Smith and Ratcliff, 2004), neural activity related to response choices, i.e., the motor plans, builds up in separate accumulators as a function of the evidence for or against them until one reaches a threshold, winning the competition.

What a motor plan is or what it represents is a very debated issue. It is generally accepted that the motor plan is formed in the premotor cortex and in M1 (Tanji and Evarts, 1976; Weinrich et al., 1984), but what is encoded by the neural activity of the motor cortices and how it relates to movement activity are matters of some controversy. It has been proposed that the discharge of single-unit might represent a subthreshold version of movement (Tanji and Evarts, 1976) or that population activity represents some movement parameters (e.g., reach direction and distance Georgopoulos et al., 1982; Riehle and Requin, 1989) or even the combination between information about the target and about the effector used (Hoshi and Tanji, 2000, 2002). However, all these approaches have been proven to be inconsistent or equivocal in fully explaining the activity in the motor system (for a review see Shenoy et al., 2013).

An alternative proposal is that this activity reflects a mix of signals: “*some will be outputs to drive the spinal cord and muscles, but many will be internal processes that help to compose the outputs, but are themselves only poorly described in terms of the movement*” (Shenoy et al., 2013, p. 339). In other words, the activity that precedes a movement would represent the initial state of a dynamic system which will determine the temporal patterns needed to drive actions. Under this hypothesis the motor plan does not explicitly represent movement parameters; it is still closely related to movement activity, but the reciprocal relationship is not transparent at the level of the individual cells. This framework seems to reconcile several past, apparently contradictory, findings and to provide a wider comprehension of the functioning of the cortical motor system (Shenoy et al., 2013). One possible limit of the dynamic system hypothesis, that future studies will have to overcome, is that most of the data come from experiments in which there is a delay between the appearance of the target and the go signal (delayed-reach task, e.g., Churchland et al., 2006). Clearly, to be fully validated the hypothesis should be tested in tasks featuring performance or inhibition of reaching movements in different contexts (but see Mattia et al., 2013).

In conclusion, the same substrates where the action selections occur are also those that are used to prepare and guide the execution of the movement that is ultimately selected.

### THE FINAL EVALUATION (LATE “SHOULD-I-STAY-OR-SHOULD-I-GO” DECISION)

By the end of the previous stages, what had been evaluated as the best action to achieve the desired goal is planned, but before the corresponding motor plan can be executed it has to pass a further final check (late “should-I-stay-or-should-I-go” decision). This is a fundamental step, because from the moment at which the decision to act has been taken to the time when the motor output is about to be generated, the continuous flow of information might signal that something has changed in the external environment, in the internal states or in both. These changes might impact on the previous evaluations as the selected action might turn out to be no longer appropriate (Haggard, 2008). A common experience might be that of a person about to cross a road but, just before stepping onto the road, he hears the sound of an ambulance siren clearly approaching. In the most common instance, the person would halt his step to avoid being hit. However, the evaluation might be radically different if his/her child is already in the middle of the road. In the former case suppressing the action is clearly the most valuable decision but, in the latter, the risk of losing the parental investment might trigger the person to act even faster in order to secure the child. The evaluation could be also influenced by endogenous signals, for instance the same person in the above example could take the risk of crossing the road because he suddenly remembers that his plane is leaving.

**FIGURE 6:**
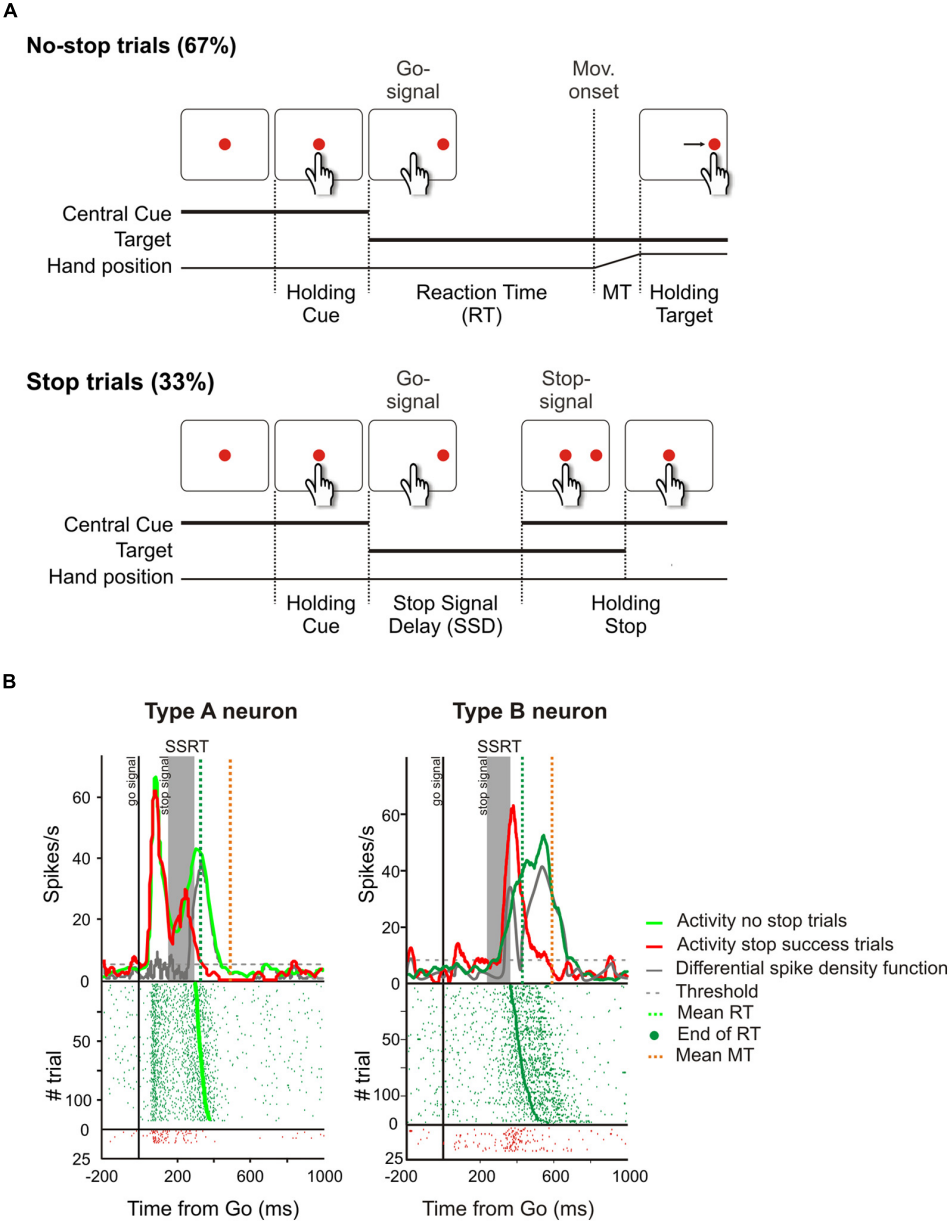
**Causal role of neurons of the dorsal premotor cortex (PMd) in reactive inhibition. (A)** Temporal sequence of the visual displays for no-stop and stop trials in the reaching version of the countermanding task. All trials began with the presentation of a central stimulus. After a variable holding delay (500–800 ms) it disappeared and simultaneously a target appeared to the right, acting as a go-signal. In the no-stop trials subjects had to start a speeded reaching movement toward the peripheral target. Randomly, on a fraction of interleaved trials (33%), the central stimulus reappeared after variable delays (SSDs), instructing subjects to inhibit movement initiation. In these stop trials, if subjects countermanded the planned movement keeping the arm on the central stimulus the trial was scored as a stop-success trial. Otherwise, if subjects executed the reaching movement the trial was scored as a stop-failure trial (not shown). Reproduced with permission from Mattia et al. (2012). **(B)** Changes of activity driven by the stop-signal onset in PMd neurons modulated during the preparation of the movement. In each panel the upper graph represents the average spike density function while the lower graph shows the raster plots of neural activity in no-stop trials (green tick-marks) and stop-success trials (red tick-marks). Neural activity was always aligned to the go-signal onset (first vertical line). The gray band represents the estimated duration of the stop-signal reaction time (SSRT) in that session. The gray line represents the differential spike density function, while the dashed gray line represents the threshold value for significant divergence. The green and the orange vertical dotted lines in the top panels indicate the average RT and the average end of MT, respectively. The green dots in the rasters represent the end of the RTs. On the right, the activity of a representative “type A” countermanding neuron is shown. In this cell, neural activity during stop-success trials (red line) initially resembles that of no-stop trials (green line) but, with a delay after the stop-signal presentation, it suddenly starts to decrease and the differential spike density function crosses the threshold 34.4 ms before the end of the SSRT. On the left, the activity of a representative “type B” countermanding neuron is shown. In this instance, the activity in stop-success trials increases after stop-signal presentation with respect to that recorded during no-stop trials 39.9 ms before the end of the SSRT. Therefore both these two types of neurons exhibit a modulation of activity sufficient to control the suppression of an ongoing arm movement.

Computationally this last check could be realized by comparing the output of a predictive forward model with a goal description (e.g., see Wolpert and Miall, 1996). When the mismatch between the predicted result and the goal becomes too large, i.e., the action is unlikely to allow the achievement of the desired result, the pending action is canceled. There are several tasks that are currently exploited to study the inhibitory function and each has some advantages over the others (for a review see Ridderinkhof et al., 2011); however, in order to design a potential neural network capable of augmenting inhibition of pending actions, I will focus on the stop-signal paradigm (Logan and Cowan, 1984). There are two reasons to choose this as a paradigmatic task. Firstly it is the only one which allows study of the suppression of ongoing movements, and secondly it has been widely used exploiting several effectors (the eyes, e.g., see Hanes and Schall, 1996; the finger, e.g., see Logan and Cowan, 1984; the arm, e.g., see Mirabella et al., 2006); thus a wealth of data are available. The stop-signal (or countermanding) paradigm probes a subject’s ability to withhold a planned movement triggered by a go signal when an infrequent stop-signal is presented after a variable delay (see **Figure 6A**).

Starting from the behavioral performance during the countermanding task it is possible to yield an estimate of the duration of the suppression process [stop-signal reaction time (SSRT); Logan and Cowan, 1984; Band et al., 2003; Boucher et al., 2007]. The SSRT is a key behavioral parameter for uncovering the neural substrates of inhibition. In fact, those brain regions showing a change in activity when a movement is produced with respect to when it is suppressed, and where the onset of this shift precedes the end of the SSRT, can be assumed to be causally related to the suppression process.

Thus the stop-signal task allows to study of the way subjects react to an unexpected imperative stop instruction. This is referred to as “reactive inhibition.” At the same time, this approach also allows assessment of changes in the response strategies of individuals embedded in such an experimental context. In fact, the rules of the countermanding task create a conflict on all no-stop trials because subjects are instructed to move as fast as possible, but, at the same time, they tend to delay movement initiation to wait for the occurrence of a possible stop-signal. As a consequence, healthy subjects had longer RTs when executing go-trials intermixed with stop-trials than when executing go-trials alone (e.g., Mirabella et al., 2006; Verbruggen and Logan, 2009). In addition, the occurrence of stop trials induces a lengthening of the RTs of responses produced in the immediately subsequent no-stop trials (Mirabella et al., 2006, 2012; Verbruggen and Logan, 2009; Zandbelt and Vink, 2010). This form of control over response execution in anticipation of known task demands, driven by endogenous signals, i.e., the awareness of the possible presentation of stop-signals, has been called “proactive control/inhibition.” In the following, I will describe results mainly related to reactive inhibition, while I will mainly focus on proactive inhibition in the next paragraph where I will deal with the monitoring system (but see also Action Execution). Clearly, all proactive strategies necessarily derive from these computations. This does not mean the neural substrates of reactive and proactive inhibition have to be different. In fact, it has been shown that there is an overlap between them (e.g., Jahfari et al., 2012; Zandbelt et al., 2013; for a review see Aron, 2011); however, reactive and proactive inhibition might derive from two conceptually different modules (the late “should-I-stay-or-should-I-go” decision module and the monitoring system; see **Figure 1**).

Variants of the stop-signal task have been used several times in association with different techniques [e.g., single-unit recordings, fMRI, electroencephalographic scalp recordings (EEG), intracranial electroencephalographic recordings (iEEF), lesions, transcranial magnetic stimulation (TMS), deep brain stimulation (DBS)], different effectors (the eyes, the arm, and the fingers), and different pathologies (e.g., PD, attention deficit and hyperactivity disorder, Tourette syndrome, obsessive–compulsive disorder). From this large number of studies, a network of brain regions that seem to be involved in implementing inhibition has been identified. To this network belong both cortical and subcortical structures, that largely overlap with those involved in movement generation, planning, and even movement execution.

One of the prefrontal regions that more frequently has been reported to have an inhibitory role is the inferior frontal gyrus (IFG), especially in the right hemisphere (see Aron et al., 2014 for a recent review). For instance, Aron et al. (2003) have shown that humans with a lesion to the right, but not to the left IFG exhibit longer SSRTs than healthy subjects. Furthermore they showed that the lengthening of the SSRT was proportional to the extent of damage in the right IFG. Less frequently, the DLPFC has also been claimed to participate in this executive function (e.g., Zheng et al., 2008). However, its role is controversial. In fact, while Zheng et al. (2008) showed that individuals who were more proficient at inhibition had a greater activation in DLPF, Chambers et al. (2006) found that temporary deactivation of the same region, with repetitive TMS, did not significantly alter the speed of inhibition.

Imagining studies revealed the involvement in this form of inhibition of the pre-SMA (e.g., Aron et al., 2007; Li et al., 2008; Zandbelt and Vink, 2010; Jahfari et al., 2012) as well as of the striatum (Li et al., 2008; Zandbelt and Vink, 2010). Even in this instance the exact role of these structures is unclear and debated. Li et al. (2008) compared the fMRI brain activation of individuals with short versus long SSRTs who were identical in all other aspects of stop-signal performance. Their aim was to isolate the neural correlates of response inhibition from those of response monitoring and/or attentional control. Under these experimental conditions, Li et al. (2008) found that the caudate head had greater activation in individuals with short than with long SSRT, and the extent of its activation was positively correlated with activity in the pre-SMA. In contrast, Scangos and Stuphorn (2010), by recording single-neuron activity of SMA and pre-SMA of monkeys during an arm countermanding task, found that these cells could not contribute directly to response inhibition as most of them modulate after the SSRT. Instead, the majority of neurons signaled expectation of reward, as they were modulated by the amount of expected reward.

Another basal ganglion implicated in stopping ongoing actions is the subthalamic nucleus (STN; see Aron and Poldrack, 2006). Mirabella et al. (2012) demonstrated that bilateral STN DBS selectively improves inhibitory functions as its electrical stimulation significantly shortened the SSRT, but did not influence the RTs of no-stop trials. These results agree with those of Van den Wildenberg et al. (2006) and Swann et al. (2011), but not with those of Li et al. (2008). In the above-described study, they found that neither the STN nor the IFG were active during reactive stop and thus concluded that both structures had a role in attentional monitoring of the stop-signal. Similarly, Zandbelt and Vink (2010) did not find evidence of STN activation during movement cancelation.

**FIGURE 7:**
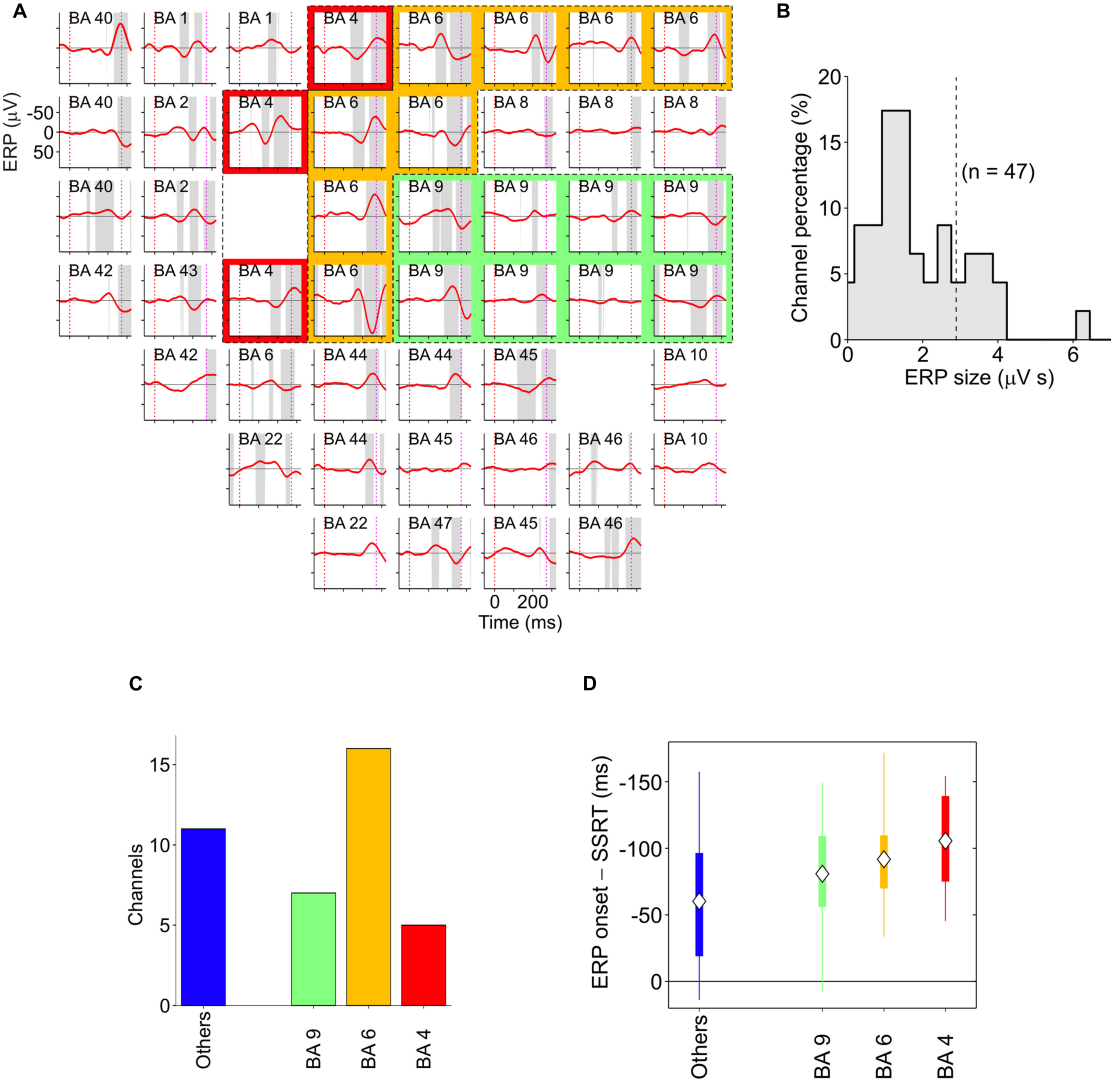
**Spatiotemporal distribution of stop-event related potentials (ERPs) in successful-stop (SS) trials. (A)** Average stop-ERPs (solid red curves) of SS trials centered on stop-signal appearance corresponding to the selected channels for one pharmacoresistant epileptic patient. Gray areas, time intervals at which the stop-ERP was significantly different from 0 (Wilcoxon signed-rank test, *P* < 0.01). Subplot labels: Brodmann areas (BAs) over which electrodes were positioned. Colored areas: electrodes placed over the primary motor cortex (red, BA4), the premotor cortex (yellow, BA6) and the DLPFC (green, BA9). **(B)** Histogram of the stop-ERP sizes of panel **(A)**. Stop-ERP sizes were computed as the integral of absolute values of stop-ERP voltage deflections in the interval periods marked by gray areas within SSRT. Dashed line: threshold value for selecting the subset of channels with large enough stop-ERPs used for population analyses (see Mattia et al., 2012 for details). **(C)** Number of channels showing large enough average stop-ERPs across five patients (*n* = 39) grouped by BA. Blue bar (others) represents those areas where channels were not selected more than twice across all patients. **(D)** Box plot of stop-ERP onsets measured with respect to the end of SSRT across all selected channels in all patients. Stop-ERP onset was defined as the first time that an electrode voltage was significantly different from 0. Diamonds indicate average onset times. Tick bars indicate the first and the third quartile. Vertical lines indicate the extreme time lags in the channel group. Freely adapted from Mattia et al. (2012), with permission.

Finally, two other areas have been found to be involved in inhibitory function, i.e., the PMd (Mirabella et al., 2011; Mattia et al., 2012, 2013) and the primary motor cortex (M1; Coxon et al., 2006; Swann et al., 2009; Mattia et al., 2012). Mirabella et al. (2011) showed that about 30% of PMd cells changed their discharge before the end of the SSRT when the monkey had to stop a reaching movement. Thus these neurons seem to be causally involved in reactive inhibition (see **Figure 6B**). These findings were confirmed and extended by a subsequent study (Mattia et al., 2012), in which epicortical event-related potentials (ERPs) were recorded from the lateral surface of the fronto-temporal lobes of epileptic patients performing the countermanding task. It was found that an ERP complex was selectively expressed before the end of the SSRT in M1, and in the premotor cortex (**Figure 7**). Thus, Mattia et al. (2012) deduced that motor cortices are causally involved in inhibitory control. In conclusion, even though the exact role of each of the brain regions involved in the stop task remains controversial, it clearly emerges a considerable overlap between brain region subserving the preparation of an action and its suppression (see **Table 1**).

To summarize, the late “should-I-stay-or-should-I-go” decision represents a hinge of our goal-directed behavior because it allows crucial, last-minute changes of strategies when the cost of an action overcomes the benefits. Unlike the early “should-I-stay-or-should-I-go” decision, which does not require active suppression, in this instance some neural signals aiming to halt the activity linked to the ongoing action have to be produced by the nervous system (**Figure 1**). Given this, it should not come as a surprise that such a large network of brain regions has to be involved.

### ACTION EXECUTION

Once the last check is passed, the motor commands are sent to the spinal cord, activate the muscles, and produce the movement. It must be remarked that it is very unlikely that even the spinal cord would be a passive relay of “higher directives.” In fact, Prut and Fetz (1999) have shown that spinal interneurons show pre-movement activity during a delayed task, similarly to PMd (Wise et al., 1983). This indicates that, at least under some experimental conditions, movement preparation may occur simultaneously over widely distributed regions, including spinal levels.

Additionally, it must be taken into account that arm movements, unlike saccades, are not ballistic movements as they can be stopped at any point along their path (De Jong et al., 1990; Scangos and Stuphorn, 2010). As a consequence, their planning can be modified even during the execution phase. This is in line with the findings of Mirabella et al. (2008), who compared RTs and movement times (MTs) of reaching movements toward visual targets executed either during an RT task (go-only task) or during a countermanding task. In both tasks subjects executed the same movements, but in the countermanding task subjects were aware that a stop-signal could randomly appear during movement preparation, indicating that the pending action should be suppressed. The awareness of the possible appearance of the stop-signal creates a conflict on all no-stop trials because, despite the instructions to always move as fast as possible, subjects spontaneously tend to delay movement initiation to wait for the possible occurrence of a stop-signal (e.g., Mirabella et al., 2006, 2012). This is a common proactive strategy that subjects implicitly adopt to have a greater chance of stopping their movements. However, the proactive strategy was also found to affect the MTs, which were shorter during the no-stop trials and longer during the go-only trials. Probably the increased length of RTs during the no-stop trials allowed subjects to fully process movement parameters and thus to move faster. In contrast, in go-only trials the absence of a proactive brake allows a shortening of RTs, at the cost of leaving some details of the motor program uncompleted, so that the planning must be completed during the movement. This strategy represents an optimization of costs versus benefits because shorter RTs are compensated by longer MTs and vice versa.

**FIGURE 8:**
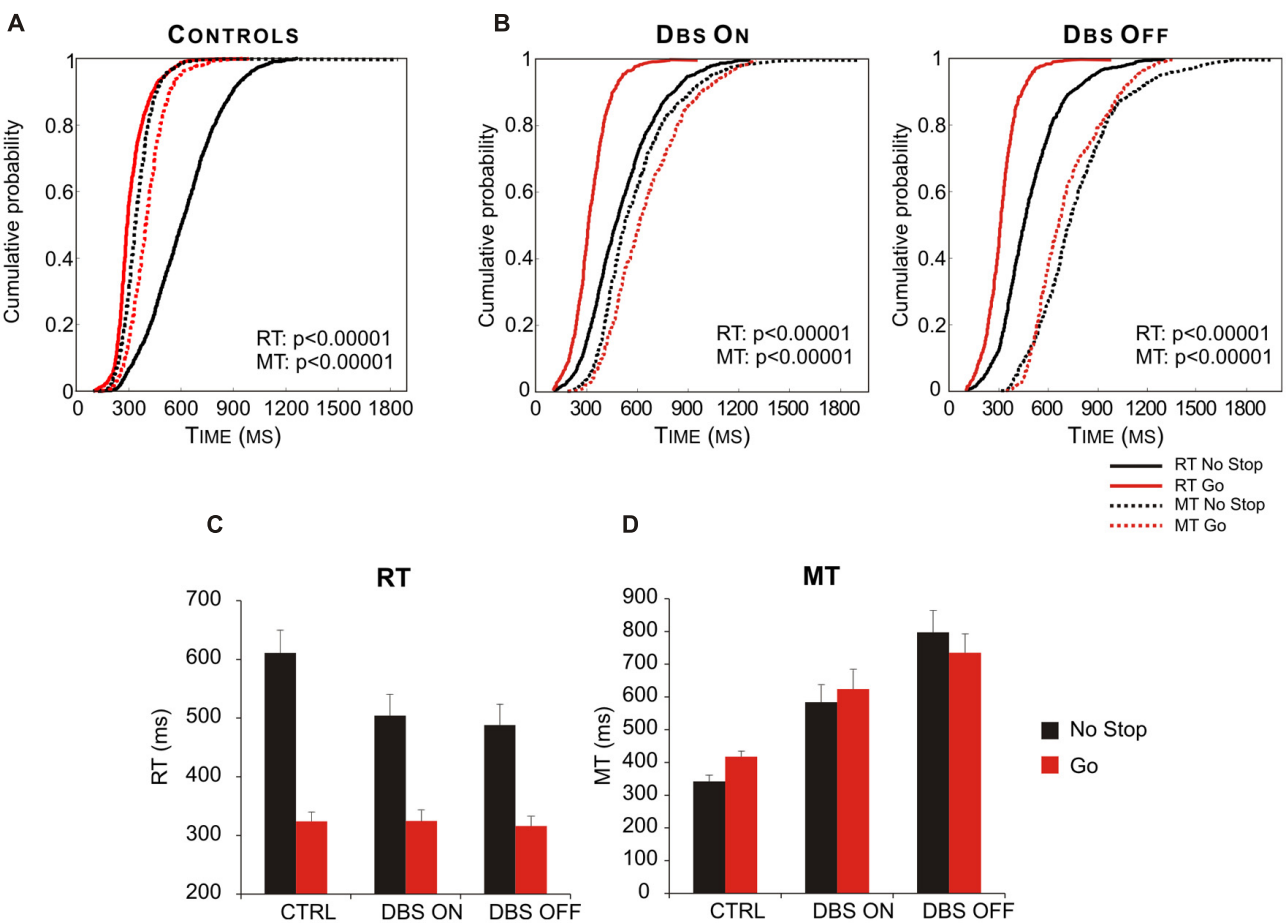
**Deep brain stimulation (DBS) of subthalamic nucleus (STN) partially restores the appropriate motor strategy according to the contexts. (A)** Cumulative distribution of RTs (solid lines) and MTs (dotted lines) of healthy subjects (*n* = 13) for go-only (red lines) and no-stop (black lines) trials. **(B)** Cumulative distributions of RTs (solid lines) and MTs (dotted lines) of DBS patients (*n*= 12) in DBS-ON and DBF-OFF conditions for both go-only (red lines) and no-stop (black lines) trials. For each condition the *P*-value of Kolmogorov–Smirnov test is given, both for RTs and for MTs. **(C)** Histograms of average RTs of no-stop and go-only trials in DBS-ON and DBF-OFF conditions. Bars represent the standard error of the mean.** (D)** Histograms of average MTs of no-stop and go-only trials in each DBS-ON and DBF-OFF condition. Bars represent the standard error of the mean. Reproduced from Mirabella et al. (2013), with permission from PLOS.

Interestingly, Mirabella et al. (2013) demonstrated that STN takes part in this process. In fact PD patients in which both DBS were turned on behaved similarly to healthy subjects, whereas when both DBS were off the same patients had both RTs and MTs longer in no-stop trials than in go-only trials (**Figure 8**). Therefore this study demonstrated the existence of a causal link between the DBS of STN and the motor strategy exploited. Once again this evidence favors the hypothesis that STN is not involved in a single function (e.g., reactive inhibition); instead, more generally, it can compute the payoff of an ongoing action.

### THE MONITORING SYSTEM

In order to learn how to make good decisions, the brain needs to compute, learn, and store the results of the outcomes that were generated by its previous decisions. To this end the ability of computing predictions about future reward and the ability of measuring discrepancies between attended and actual outcomes are fundamental. These functions are performed by a set of brain regions that collectively I will call the “monitoring system” (**Figure 1**). This system is composed of a set of cortical and subcortical structures that allow coding of reward expectations, detection of errors and implementation of behavioral adjustments, aiming to cope with more or less demanding context in order to optimize future choices. Therefore signals produced by the monitoring system can influence any stage of action implementation.

**FIGURE 9:**
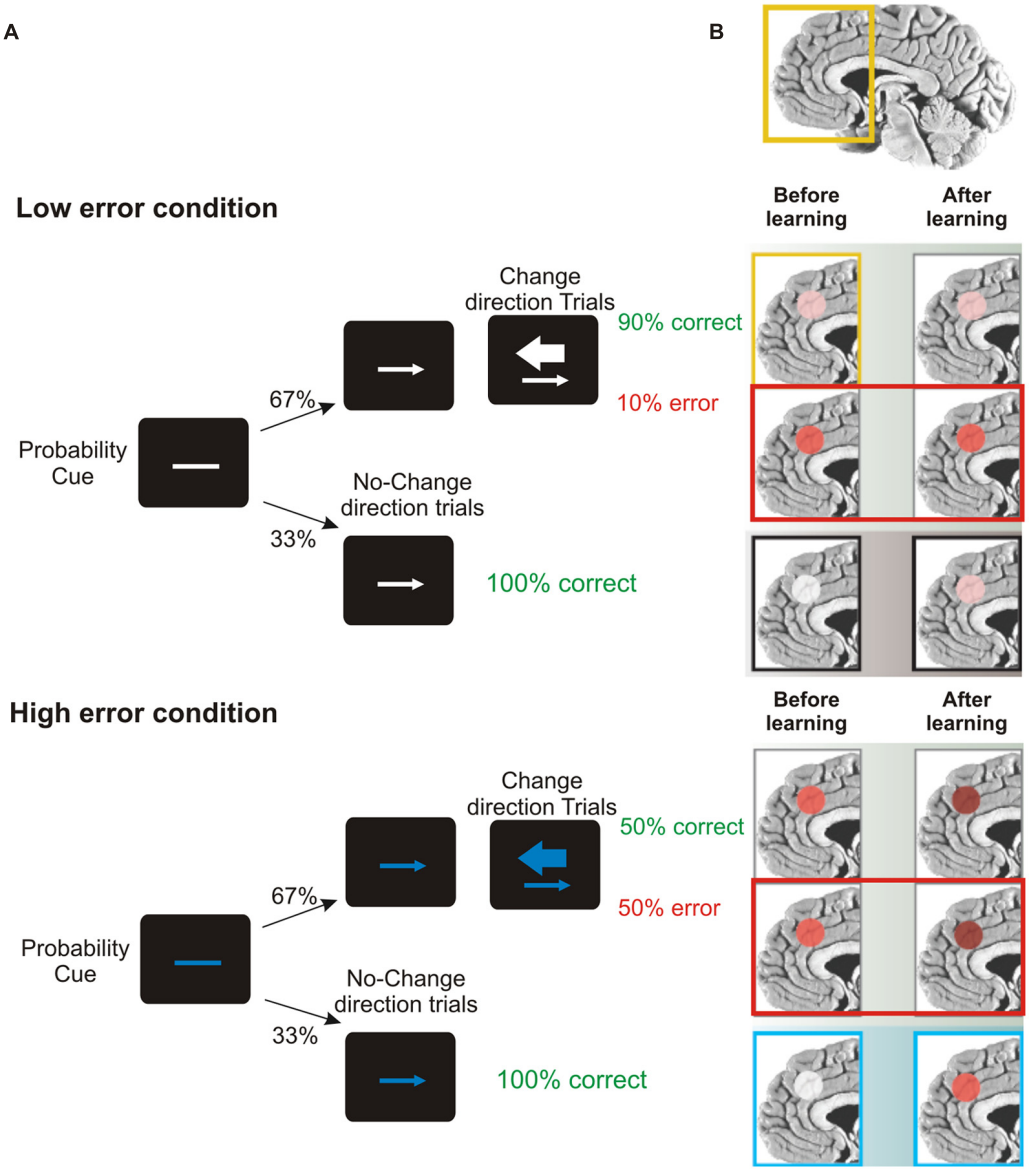
**Role of ACC in predicting error likelihood. (A)** Temporal sequence of the visual displays for the change-signal task. Initially a probability cue (plain line) was displayed. The cue could be either white or blue, predicting low or high error likelihood respectively. After a delay, a go signal was presented (left or right pointing arrow) indicating the required button-press response (left or right index press for left or right pointing arrow, respectively). Randomly on 33% of the trials a change signal was displayed (a larger arrow pointing in the opposite direction with respect to that presented as go signal). To this signal, subjects had to reverse the response from that indicated by the go signal. Error rates were controlled by a staircase procedure so that in the low error condition the delay between the go signal and the change signal was kept shorter and subjects made around 90% of correct responses. In the high error condition the delay was kept longer and subjects made around 50% correct responses. **(B)** Brain regions highlighted indicate the activation of ACC during the stop-change before and after learning (the greater the activation, the deeper the red color) for correct, wrong change-direction trials and for no-change trials (for the top to the bottom row) in the low and high error conditions. Interestingly, during no-change trials ACC activity increased with practice, especially in response to blue color cues, reflecting an improved ability to predict the likelihood of making an error. Freely adapted from Ridderinkhof and van den Wildenberg (2005), with permission.

A key role in this set of processes is played by the ACC, located in the frontal medial wall (**Figure 3**). It has been known for a long time that this area is involved in cognitive control over behavior (e.g., Bernstein et al., 1995; Botvinick et al., 1999); however, its precise role remains considerably debated. One influential hypothesis is that the activity of ACC might represent the likelihood of obtaining or losing reward in response to particular actions. Evidence in support of this idea comes from the results of Brown and Braver (2005). They administered to human volunteers a stop-change task while recording the fMRI BOLD signal. The task, (**Figure 9**), required participants to make rapid left- or right-hand button-press responses according to the direction of an arrow. In 33% of cases, during the RTs, a second arrow pointing in the opposite direction was presented and the responses had to be reversed. Using a staircase algorithm, authors adjusted the time of presentation of the second arrow, hence controlling the likelihood of making mistakes (either 10 or 50%). At the beginning of each trial, participants were informed of the error likelihood by a color cue: a blue bar indicated 50% error probability, while a white bar indicated 10% error probability. The activity of ACC was greater during the high error than during the low error conditions. This held true not only during change trials, but also during trials in which participants did not have to change the responses (**Figure 9**). Therefore ACC does not simply indicate response conflict (occurring when the two arrows are presented) or error occurrence; instead more generally it signals the perceived likelihood of potential errors. Crucially, Brown and Braver (2005) exploiting a neural-network model, showed that this pattern of response gradually emerged over the course of the experimental session, i.e., after individuals learned the associations between color cue and error likelihood.

Other studies point to a slightly different role for the ACC. According to these accounts, ACC would provide a continuously updated prediction of expected cognitive demand which will be used to optimize future behavior. For instance, Sheth et al. (2012) showed that ACC activity speeds up behavioral responses when cognitive demand remain stable, but, in more challenging situations, it slows down responses to allow a greater accuracy. Kennerley et al. (2006), studying the behavioral responses of monkeys before and after ACC lesions, confirmed that this region adaptively guides future behaviors, but exploiting a different mechanism. In a key experiment, monkeys were rewarded when performing a certain action (e.g., lifting a lever) until the rewarded action was changed (e.g., to get the reward it had to turn the lever). Non-lesioned monkeys had no difficult in this task, while ACC-lesioned monkeys following an unrewarded lift response switched to turning, but could not sustain this response on subsequent trials. Thus, the lesion compromised the ability to associate payoffs with the outcome of past actions to adaptively guide future behavior.

Whatever the exact role of ACC in monitoring behavioral performance, it appears that this capability develops through experience. It is very probable that the knowledge of past experiences is built through reinforcement-learning processes, mediated by the discharge of midbrain dopamine neurons. The discharge of these neurons measures deviations from individuals’ previous reward expectations, i.e., they compute the so-called prediction errors (Schultz et al., 1997; O’Doherty et al., 2003; Bayer and Glimcher, 2005). Every time an event is better than expected, dopaminergic neurons phasically increase their discharge. In contrast, they do not change firing rate when an event occurs as predicted, while they decrease their discharge if something worse than expected takes place. Prediction errors are thought to play a key role in guiding decision-making by signaling the need to adjust future behavior, i.e., they are fundamental to learning processes (Schultz et al., 1997; Montague et al., 2004).

Dopaminergic neurons project widely to the striatum and to several regions of PFC including the ACC (Freund et al., 1984; Smith et al., 1994). The activity of ventral striatum seems to correlate with prediction error computation (Bray and O’Doherty, 2007; Hare et al., 2008) and thus it is likely to contribute to some further elaboration of action-outcome predictions. However, ACC plays a different role from ventral striatum, exploiting prediction errors as training signals to build extended action-outcome histories, that later could be exploited to adapt goal-directed behaviors (Holroyd and Coles, 2002; Brown and Braver, 2005; Kennerley et al., 2006). Given such a complex function, it is unlikely that ACC would rely solely on signals coming from dopaminergic neurons. In fact, other brain regions have been shown to produce signals promoting learning. For instance, it has been shown that neurons of the frontal pole cortex code the outcome of actions (Tsujimoto et al., 2010; for a review see Tsujimoto et al., 2011). Also, neurons in SMA and pre-SMA of monkeys have been shown to be modulated by the amount of expected reward and thus these regions might, among other things, be signaling expectation of reward too (Scangos and Stuphorn, 2010). Not all studies point to this conclusion, e.g., recently Bonini et al. (2014), by recording evoked field potentials in pharmacoresistant epileptic patients, claimed that SMA continuously assesses ongoing actions and, when an error occurs, it signals to ACC.

Whatever the exact role of each of these components, they form a system that is capable of monitoring actions, evaluating their behavioral outcomes, and learning the association between a certain environmental context and the likelihood that a certain action or strategy will lead to the desired goal. Thus, the monitoring system is the ideal candidate for implementing proactive preparation of action plans in anticipation of known task demands. Such advance preparation has been studied in the context of response inhibition, in particular when the countermanding task is employed. This is because the rules of this paradigm produce a conflict every time subjects have to execute no-stop trials (see **Figure 6A**). As described above, this context induces a lengthening of the RTs and at the same time a shortening of MTs with respect to situations in which the same movements have to be performed, but stop-signals are never presented (e.g., Mirabella et al., 2008, 2013).

The study of the neural underpinnings of proactive control revealed that the network of brain regions that subserve this function largely overlaps with those subserving reactive inhibition. This conclusion stems from several fMRI studies which employed a probabilistic stop task with cues indicating the likelihood of stop trial occurrence (e.g., Chikazoe et al., 2009; Zandbelt and Vink, 2010; Jahfari et al., 2012; Zandbelt et al., 2013). Some cues indicate that go-signals are never followed by a stop-signal (certain go-signals), whereas others indicate that go-signals have a certain likelihood of being followed by a stop-signal (uncertain go-signals). By comparing brain activity between these conditions, it has been possible to uncover the neural substrate of advanced action preparation. Overall it emerged that when subjects expect the occurrence of a stop-signal, they proactively engage the network subserving reactive inhibition in a progressively increasing fashion according to the likelihood of stop presentation. A lower stop probability would correspond to a weaker activation of the network and to less marked slowing of responses, while when the stop is presented the activation is much stronger and the motor output is completely blocked (Aron, 2011).

These studies did not show clear activations of either of ACC or midbrain dopaminergic neurons in the ventral tegmental area and in the substantia near pars compacta (but see Zandbelt et al., 2013). However, these might be due either to the fact that commonly a region-of-interest approach had been chosen or because of limitations of the fMRI technique. Nevertheless, the activation of other components of the monitoring system, i.e., striatum, SMA and pre-SMA, has been shown several times (Chikazoe et al., 2009; Zandbelt and Vink, 2010; Jahfari et al., 2012; Zandbelt et al., 2013). This activity might reflect the computations which underlie learning of the association between the experimental context and the likelihood of performing (or stopping) an action. Thus, these signals should drive the proactive network so as to maximize the probability of moving without missing stop trials. Further studies are needed to test this hypothesis.

## CONCLUDING THOUGHTS

On the whole, it seems clear that the neural network subserving goal-directed actions is very extensive, encompassing both frontal and parietal area as well as subcortical brain structures. This does not come as a surprise, as we live in a complex and ever-changing environment, which continuously offers an enormous variety of opportunities for potential actions. Thus, our motor system is called on to perform a continuous evaluation of alternative actions that may become available, in order to decide whether to persist in a given activity or to stop it and switch to a different one. This is the hinge of our behavioral flexibility. Central to this feature are the abilities of predicting the likelihood of achieving the desired goal with a certain action or strategy, and of canceling pending actions when they are no longer valuable. These two processes operate continuously during the entire course of a movement, during its genesis, its planning, and even its execution, so that the motor output can be modulated or suppressed at any time before its execution.

These considerations are clearly at odds with the old-fashioned serial framework according to which “*we sense the world, think about it, and then act upon it*” (Cisek and Kalaska, 2010, p. 271). Instead, they are compatible with the idea of parallel processing. Thus, one or more goals or actions can be coded at the same time, so that alternatives can be ready for release at short notice. Importantly, the evaluation of an action can also lead to its suppression when it has already been selected. Very probably the neural machinery underling action preparation and the neural substrates underling action inhibition are simultaneously active. From what is stated above, it is not too surprising that many of the regions mediating the decisions to act appear to be involved also in decisions to refrain from acting (Coxon et al., 2006; Mirabella et al., 2011; Mattia et al., 2012; see **Table 1**). This leads to the main point of the present review. Many, if not all, brain regions belonging to the network which controls goal-oriented movements are involved in more stages of this process. For instance, the SMA and the pre-SMA are involved in the early “should-I-stay-or-should-I-go” decision as they are involved in gating action affordances (e.g., Sumner et al., 2007; Ridderinkhof et al., 2011). However, they are also part of the monitoring system (see The Monitoring System) and they contribute to the late “should-I-stay-or-should-I-go” decision (Aron and Poldrack, 2006; Aron et al., 2007). In addition it has been shown that stimulation of SMA generates a feeling of an urge to move a particular body part, without necessarily causing any actual movement (Fried et al., 1991). The involvement of SMA in the feeling of volition has recently been confirmed by Fried et al. (2011) who recorded single-neuron activity in epileptic pharmacoresistant patients. Both PMd and M1 are involved both in the genesis of motor plans (Tanji and Evarts, 1976; Weinrich et al., 1984; Hoshi and Tanji, 2000, 2002; Churchland et al., 2006; Mattia et al., 2013; Shenoy et al., 2013) and also in their inhibition (Coxon et al., 2006; Mirabella et al., 2011; Mattia et al., 2012). Most of the network subserving reactive inhibition is also involved in proactive inhibition (see Action Execution).

Some other brain regions are involved not solely in motor controls, but also in very different tasks. The ACC has been shown to be involved in monitoring behavioral performance (see The Monitoring System), but also in pain processing (Singer et al., 2004). The role of the IFG is even more controversial as it seems to perform several different functions, some of which are lateralized (see Liakakis et al., 2011 for a review). The left IFG is implicated in both perceptive and productive aspects of language (see Uddén and Bahlmann, 2012 for a review). Some studies indicate that the right IFG plays a key role in redirecting selective attention toward unexpected stimuli (Corbetta and Shulman, 2002); others suggest that it has a specific role in motor inhibition (Aron et al., 2014) or in suppression of unwanted memories (Benoit and Anderson, 2012). In addition, the IFG of both hemispheres are involved in processing of symbolic gestures used for social non-verbal communication (Lindenberg et al., 2012), and they are regions belonging to the core of the human mirror-neuron system, i.e., of the system that is thought to allow the ability of understanding the intentions of others (for a review see Rizzolatti and Craighero, 2004). Finally, the PFC contribute to an enormous array of functions (for a review see Wise, 2008).

Given this picture, I believe that is rather hard to assign a very specific role to most of those regions when performing complex cognitive functions, such as the augmentation of adaptive behaviors. Possibly, complex cognitive functions are not performed by a unique structure. Rather, they might emerge from the coordinated activity of large-scale neuronal networks that are dynamically configured on fixed anatomical connections (von der Malsburg et al., 2010). The mechanisms underlying the dynamic coordination of neural populations are poorly understood. Nevertheless, it has been suggested that the formation of functional networks is achieved by modulating the degree of coherence among temporally structured responses of widely distributed neurons. In its turn, coherence might be modulated by rhythmic modulation of activity and synchronization among these populations (see Fries, 2005 for a review). According to this framework a given brain region can perform specific operations, e.g., the right IFG might subserve the reorienting of selective attention (Corbetta and Shulman, 2002) or the ACC might compute the likelihood of a successful action, but its functional features can be exploited in a variety of tasks. Thus the outcome of complex processes would depend not upon the activation of one or a few brain regions, but on the specific interactions of the network activated during the ongoing task. This hypothesis would explain why the same regions are active under many different contexts, and it might also help to understand the limited success achieved by brain–machine interfaces (BMIs; for reviews see Baranauskas, 2014; Bensmaia and Miller, 2014).

The large majority of BMI interfaces are aimed at restoring body mobility in patients suffering from motor deficits caused by brain injury, neurologic diseases and limb loss (Bensmaia and Miller, 2014). BMIs record brain signals, decode movement-related information and use these to control external devices (e.g., prosthetic limbs, wheelchairs). Although at present the BMI approach had been fruitful (e.g., Hatsopoulos and Donoghue, 2009) it has also shown several limitations. Patients have to undergo a long period of training before learning how to use the BMIs, and signals extracted from the brain are noisy and difficult to interpret so the error rate is rather high and the response speed is slow. All in all, the guidance of external devices is still far from approximating natural behaviors. This applies to both non-invasive and invasive BMIs. Non-invasive BMIs are mainly based on EEG signals recorded from the scalp. Activity recorded at the scalp level lacks selectivity; it is made up of a mix of signals coming from different cortical regions and thus the amount of useable information conveyed is likely to be too low (Baranauskas, 2014). On the other hand, invasive BMIs employ multiple electrodes surgically inserted into the brain (usually in M1 and/or the somatosensory cortex). Even though the quality of the recorded signals is definitely better, information transfer rate is still not dramatically improved (Baranauskas, 2014). The BMI approach has been shown to reproduce relatively simple behaviors such as two-dimensional center-out reaching tasks (e.g., Wolpaw and McFarland, 2004). Other much more complex tasks, e.g., those entailing suppression of pending actions (Mirabella, 2012), have almost never been tested (but see Ifft et al., 2012). The relatively low degree of success of invasive BMI might be due to the fact that, due to ethical reasons, it has been possible to capture only a very limited amount of the activity of the neural network underling goal-directed actions. However, as stated above, flexible control of behavior can be achieved only through the functional interactions of several brain regions, so it is possible that signals recorded from a limited cortical area fail to provide enough information to reproduce natural movements. To overcome these limitations, it would be necessary to find a way to record simultaneously from several key regions underlying goal-directed actions. Clearly, this is currently not feasible on humans beings, though it could be tested on non-human primates.

Before concluding, I would like to make an important remark: all processes described in the present review, i.e., genesis and suppression of goal-directed actions, do not require either intention, volition, or awareness. These computations can be performed by most animals, and surely by all primates. Not by chance, many of the data described here come from studies on non-human primates. What exactly volition or free will is and how and when it is generated from brain activity is, at best, unclear. There is a consensus around the idea originally proposed by Libet et al. (1983) that the awareness of intention precedes movements by some 100s of milliseconds. Thus, it is possible that the brain generates a action and, only subsequently, some pre-motor processes produce the subjective experience of willing to execute that action, which is perceived as being freely chosen (Hallett, 2007). To overcome the impasse of these findings, Libet (1985) proposed that because awareness of intention precedes movements, our free will should rely on the ability to withhold upcoming actions (see Mirabella, 2007; Haggard, 2008 for reviews). However, it has been shown that action suppression can be implemented unintentionally and unconsciously (see van Gaal et al., 2008). Furthermore, both monkeys (e.g., Mirabella et al., 2011) and rats (e.g., Eagle et al., 2008) can cancel pending movements. It has to be recognized that most studies on action inhibition in humans rely on externally and not on internally triggered stops (see Brass and Haggard, 2007 for an original approach), so further evidence has to be collected before venturing conclusions around the relationship between the veto power and willingness. As Roskies (2010) stated: “*For now, […], the most significant contribution neuroscience has made has been in allowing us to formulate novel questions about the nature of voluntary behavior, and in providing new ways of addressing them.*” I hope that the present review will also help to circumscribe what free will is not.

## Conflict of Interest Statement

The author declares that the research was conducted in the absence of any commercial or financial relationships that could be construed as a potential conflict of interest.

## Footnotes

1 An exception to this model might occur when the number of potential goals exceeds the capacity of working memory. In these instances goals might be processed differently.

2 As previously stated for the representation of multiple goals, even in this instance the representation of multiple potential movements may be limited by the capacity of working memory.
